# Vitamin D: a critical and essential micronutrient for human health

**DOI:** 10.3389/fphys.2014.00248

**Published:** 2014-07-11

**Authors:** Igor Bendik, Angelika Friedel, Franz F. Roos, Peter Weber, Manfred Eggersdorfer

**Affiliations:** Human Nutrition and Health (IB, AF, FFR), and Nutrition Science & Advocacy (PW, ME), DSM Nutritional Products Ltd.Basel, Switzerland

**Keywords:** vitamin D, 25-hydroxyvitamin D, nutrition, micronutrients, hidden hunger, nutrition security, nutritional pathways, nutrigenomics

## Abstract

Vitamin D is a micronutrient that is needed for optimal health throughout the whole life. Vitamin D_3_ (cholecalciferol) can be either synthesized in the human skin upon exposure to the UV light of the sun, or it is obtained from the diet. If the photoconversion in the skin due to reduced sun exposure (e.g., in wintertime) is insufficient, intake of adequate vitamin D from the diet is essential to health. Severe vitamin D deficiency can lead to a multitude of avoidable illnesses; among them are well-known bone diseases like osteoporosis, a number of autoimmune diseases, many different cancers, and some cardiovascular diseases like hypertension are being discussed. Vitamin D is found naturally in only very few foods. Foods containing vitamin D include some fatty fish, fish liver oils, and eggs from hens that have been fed vitamin D and some fortified foods in countries with respective regulations. Based on geographic location or food availability adequate vitamin D intake might not be sufficient on a global scale. The International Osteoporosis Foundation (IOF) has collected the 25-hydroxy-vitamin D plasma levels in populations of different countries using published data and developed a global vitamin D map. This map illustrates the parts of the world, where vitamin D did not reach adequate 25-hydroxyvitamin D plasma levels: 6.7% of the papers report 25-hydroxyvitamin D plasma levels below 25 nmol/L, which indicates vitamin D deficiency, 37.3% are below 50 nmol/Land only 11.9% found 25-hydroxyvitamin D plasma levels above 75 nmol/L target as suggested by vitamin D experts. The vitamin D map is adding further evidence to the vitamin D insufficiency pandemic debate, which is also an issue in the developed world. Besides malnutrition, a condition where the diet does not match to provide the adequate levels of nutrients including micronutrients for growth and maintenance, we obviously have a situation where enough nutrients were consumed, but lacked to reach sufficient vitamin D micronutrient levels. The latter situation is known as hidden hunger. The inadequate vitamin D status impacts on health care costs, which in turn could result in significant savings, if corrected. Since little is known about the effects on the molecular level that accompany the pandemic like epigenetic imprinting, the insufficiency-triggered gene regulations or the genetic background influence on the body to maintain metabolic resilience, future research will be needed. The nutrition community is highly interested in the molecular mechanism that underlies the vitamin D insufficiency caused effect. In recent years, novel large scale technologies have become available that allow the simultaneous acquisition of transcriptome, epigenome, proteome, or metabolome data in cells of organs. These important methods are now used for nutritional approaches summarized in emerging scientific fields of nutrigenomics, nutrigenetics, or nutriepigenetics. It is believed that with the help of these novel concepts further understanding can be generated to develop future sustainable nutrition solutions to safeguard nutrition security.

## Introduction

Vitamin D is needed to maintain calcium concentrations within a narrow physiological range. This function is vital as the calcium ion is essential for a large variety of cellular and metabolic processes in the body (Berridge, 2012). To secure the calcium supplies besides intestinal absorption, calcium is stored in the skeleton and acts as a large calcium reservoir that is mainly controlled by PTH and vitamin D (Bouillon et al., 2014). Humans produce vitamin D by exposure to sunlight that includes ultraviolet B radiation; if ultraviolet B radiation is not available in sufficient amounts, vitamin D needs to be obtained from the diet or dietary supplements (Holick, 2007). The start of the vitamin D endocrine system is believed to have been initiated before the start of vertebrates and evolved over millions of years (Bouillon and Suda, 2014). Therefore, the vitamin D micronutrient either synthesized through the sun by the skin or through dietary uptake is well-adapted to the human body. The endogenously conjugated vitamin D metabolites have taken over many important roles in the maintenance of human health, of which many still await to be discovered.

In this paper, we summarize the knowledge on vitamin D as an essential micronutrient important for human health and discuss the new nutritional research on its way to gain further knowledge on the function of vitamin D for nutrition.

## Vitamin D part of nutrition and content in foods

The history of vitamin D is linked to first scientific description of the classic bone disease rickets by Whistler in 1645 (Norman, 2012). Two centuries later it was Schütte who observed the usefulness of cod liver oil in the treatment of rickets and osteomalacia in 1824. The hunt for the anti-rachitic factor ended in early twentieth century, when Mellanby could demonstrate in a series of hallmark studies (1919–1924) that a nutritional component in the diet was the anti-rachitic factor to prevent rickets (Mellanby, 1919, 1976; Platt, 1956). Shortly after, vitamin D was inaugurated without the characterization of the chemical structure. In 1919, Hudschinsky showed in parallel that UV light was able to ameliorate rickets by increasing calcification in rachitic children (Huldschinsky, 1919, 1926). Both findings of the cod liver oil and the UV light preventing rickets remained independent observations until Hess and Weinstock elegantly could demonstrate that the anti-rachitic vitamin D was produce by UV irradiation in skin (Hess and Weinstock, 1925a,b). In 1936, Windaus and colleagues determined the chemical structure of the fat-soluble seco-steroid vitamin D (Windaus et al., 1936).

The vitamin D definition comprises a group of molecules called the calciferols. The main forms present in foods are cholecalciferol (vitamin D_3_) and ergocalciferol (vitamin D_2_), whereas the metabolite 25-hydroxycholecalciferol (25-hydroxyvitamin D_3_) is a natural part of the food chain by its occurrence in animal products. Vitamin D_3_ is unique by the fact that the same nutrient can be synthesized in the skin through the action of sunlight or being taken up by diet. This dual source of intake secures the body to maintain sufficient vitamin D levels in the body. The production in skin is usually the major vitamin D_3_ source for the body. However, in countries that receive insufficient sun exposure, people rely on dietary vitamin D as a major source. Exposure of the precursor 7-dehydrocholesterol in the basal and suprabasal layers of the epidermis to ultraviolet B (UVB) light with a wavelength of 290–315 nm is needed for the formation of the previtamin D_3_. The subsequent conversion is a non-enzymatic process that includes a thermal isomerization of the previtamin D_3_ to produce vitamin D_3_ (Collins and Norman, 2001; Holick, 2011). This vitamin D_3_ is rapidly converted to 25-hydroxyvitamin D_3_ in the liver. The vitamin D status is evaluated by measuring the circulating levels of serum 25-hydroxyvitamin D, which is the sum of cutaneous synthesis (vitamin D_3_) or dietary contribution (vitamin D_3_ and vitamin D_2_). The 25-hydroxyvitamin D_3_ needs to be further hydroxylated in the kidney (or locally in other organs Lehmann et al., 2001) to form 1,25-dihydroxyvitamin D_3_, the active endogenous hormone, which is responsible for most of the physiological actions of vitamin D through the binding to the vitamin D receptor (VDR). The plant-derived vitamin D_2_ is processed in the same way. For both vitamers, vitamin D_2_, and vitamin D_3_, the consecutive molecular action is believed to be identical, whereas only 1,25-dihydroxy vitamin D_3_ is the endogenous hormone, the activated vitamer 1,25-dihydroxyvitamin D_2_ is hormone mimetic. Therefore, it was not surprising that vitamin D_3_ has been reported to be superior to vitamin D_2_ in terms of bioavailability and maintaining the vitamin D status by the majority of studies (Trang et al., 1998; Armas et al., 2004; Romagnoli et al., 2008; Glendenning et al., 2009; Heaney et al., 2011; Lehmann et al., 2013). Only one study reported that the two vitamers were essentially equipotent (Holick et al., 2008).

The level of cutaneous vitamin D_3_ synthesis is mainly affected by the amount of solar UVB radiation reaching the human skin, which is a function that needs to take into account the wavelength, thickness of the ozone layer in the atmosphere and solar zenith angle. Furthermore, the geographic latitude, season of the year and time of day influence and restrict the skin-borne synthesis of vitamin D_3_ (Webb et al., 1988; Holick, 2011). It was described that vitamin D_3_ synthesis in the skin declines with age, which is due in part to a fall of 7-dehydrocholesterol and the morphological changes due to biological aging (MacLaughlin and Holick, 1985; Holick et al., 1989). Matsuoka et al. (1991) have shown that in Caucasians and Asian subjects having a lighter skin pigmentation UVB radiation produce significantly higher vitamin D_3_ serum levels than in African American and East Indian groups. It is not of a surprise that skin pigmentation reduces vitamin D_3_ formation. This skin tone dependent down regulation is easily overcome by increased sun exposures (Armas et al., 2007). Apart to darker pigmented skin, cutaneous vitamin D_3_ production can be reduced for many other reasons like severe air pollution in large cities, less outdoor activity as a consequence of an unhealthy lifestyle change, immobility of institutionalized elderly populations, topical application of sunscreens with a high sun protection factors or cultural dress codes (e.g., veiling). Therefore, dietary intake of vitamin D through foods or supplements plays a vital part to maintain healthy vitamin D levels.

Through nutrition, vitamin D intake is limited. There are few naturally-occurring food sources containing relevant levels of vitamin D. Table 1 summarizes the vitamin D content in selected foods. Vegetarian diets are limited to the plant vitamin D_2_ that is only present in some mushrooms. Commercially dark cultivated white button mushrooms contain low amounts of vitamin D_2_, only wild mushrooms or sun-dried mushrooms contain elevated amounts of ergocalciferol (Mattila et al., 1994, 1999b, 2001; Teichmann et al., 2007). Some commercial producers include an UVB radiation step to increase the vitamin D_2_ content in their products (Mau et al., 1998; Roberts et al., 2008). Vitamin D_2_ is formed out from ergosterol in the mushrooms. Some plants that are used as foods however can contain ergosterol, but this provitamin form is not converted to vitamin D_2_. Vitamin D_3_ is not found in food-borne plants. In plants, the occurrence of vitamin D_3_-related compounds is scarce. Interestingly, species belonging to the botanical *Solanaceae* family, like *Solanum malacoxylon* (*Solanum glaucophyllum* and *Solanum glaucum*), contain a glycoside of the active 1,25-dihydroxyvitamin D_3_ hormone (Boland, 1986; Boland et al., 2003; Japelt et al., 2013). This deciduous shrub (1.5–3.0 m stem length) is widely distributed in the provinces of Buenos Aires in Argentina and in Brazil and is responsible for the calcinotic disease in cattle and other grazing animal.

**Table 1:** **Vitamin D content in raw products, processed foods, and fortified foods**.

**Category**	**Foodstuff**	**Range**		**References**
		**(μg vitamin D per 100 g)**	**(IU vitamin D per 100 g)**	
**RAW PRODUCTS**				
Fish	Herring	2.2–38.0	88–1,520	Kobayashi et al., 1995; Mattila et al., 1995a, 1997; Ostermeyer and Schmidt, 2006; Byrdwell et al., 2013
	Salmon	4.2–34.5	168–1,380	Kobayashi et al., 1995; Ostermeyer and Schmidt, 2006; Lu et al., 2007; Byrdwell et al., 2013
	Halibut	4.7–27.4	188–1,094	Ostermeyer and Schmidt, 2006; Byrdwell et al., 2013
	Perch	0.3–25.2	12–1,012	Mattila et al., 1995a, 1997; Ostermeyer and Schmidt, 2006; Byrdwell et al., 2013
	Trout	3.8–19.0	152–760	Mattila et al., 1995a; Ostermeyer and Schmidt, 2006; Byrdwell et al., 2013
	Tuna	1.7–18.7	68–748	Takeuchi et al., 1984, 1986; Kobayashi et al., 1995; Byrdwell et al., 2013
	Mackerel	0.5–15.5	20–620	Egaas and Lambertsen, 1979; Aminullah Bhuiyan et al., 1993; Kobayashi et al., 1995; Ostermeyer and Schmidt, 2006; Lu et al., 2007
	Cod	0.5–6.9	20–276	Kobayashi et al., 1995; Mattila et al., 1995a; Ostermeyer and Schmidt, 2006; Byrdwell et al., 2013
Mushrooms	Morel	4.2–6.3	168–252	Phillips et al., 2011
	Dark cultivated white bottom mushrooms	0–0.2	0–8	Mattila et al., 2001; Teichmann et al., 2007; Phillips et al., 2011
	Wild grown mushrooms	0.3–29.8	10–1,192	Mattila et al., 1994, 1999b, 2001; Kobayashi et al., 1995; Teichmann et al., 2007
Animal products	Pork meat	0.1–0.7	4–28	Kobayashi et al., 1995; Bilodeau et al., 2011; Strobel et al., 2013
	Beef meat	0–0.95	0–38	Kobayashi et al., 1995; Montgomery et al., 2000, 2002; Bilodeau et al., 2011; Strobel et al., 2013
	Chicken meat	0–0.3	0–12	Kobayashi et al., 1995; Mattila et al., 1995b; Bilodeau et al., 2011; Strobel et al., 2013
	Beef liver	0–14.1	0–560	Kobayashi et al., 1995; Mattila et al., 1995b; Montgomery et al., 2000, 2002
	Eggs	0.4–12.1	28–480	Mattila et al., 1992, 1999a; Kobayashi et al., 1995; Bilodeau et al., 2011; Exler et al., 2013
**PROCESSED FOODS**				
Fish	Tuna (skipjack) liver oil	144,400	5,776,000	Takeuchi et al., 1984
	Halibut liver oil	13,400	536,000	Egaas and Lambertsen, 1979
	Cod liver oil	137.5–575.0	5,500–23,000	Egaas and Lambertsen, 1979; Takeuchi et al., 1984
	Canned pink salmon	12.7–43.5	508–1,740	Bilodeau et al., 2011
	Canned sardines	3.2–10	128–400	Mattila et al., 1995a
	Smoked salmon	4.9–27.2	196–1,088	Ostermeyer and Schmidt, 2006
Mushrooms	Irradiated mushrooms	6.6–77.4	264–3,094	Mau et al., 1998; Roberts et al., 2008
Dairy	Butter	0.2–2.0	8–80	Kobayashi et al., 1995; Mattila et al., 1995b; Jakobsen and Saxholt, 2009
	Cheese	0–0.1	0–4	Mattila et al., 1995b; Wagner et al., 2008
**FORTIFIED FOODS**				
Cereals	Corn flakes	2–4.7	87–189	Haytowitz et al., 2009; U.S. Department of Agriculture, 2013
Beverages	Orange juice	1.1	44	Wacker and Holick, 2013
	Malted drink mix, powder	3	123	Haytowitz et al., 2009; U.S. Department of Agriculture, 2013
Dairy	Milk	1.1–2.0	42–79	Calvo et al., 2004; Haytowitz et al., 2009; U.S. Department of Agriculture, 2013
	Cheese	2.6–25.0	102–1,000	Haytowitz et al., 2009; Tippetts et al., 2012; U.S. Department of Agriculture, 2013

Animal food products are the main dietary source for naturally occurring vitamin D_3_ (Schmid and Walther, 2013). Since the discovery of vitamin D, vitamin D was associated with oily fish products. It was driven by the early observation that the amount of vitamin D in a teaspoon of cod liver oil was sufficient to prevent rickets in infants. It is still the fish liver oil that contains the highest amounts of vitamin D_3_. The highest reported concentration was found in skipjack liver oil 144,400 μg/100 g (Takeuchi et al., 1984). The fish liver oils besides other nutritional ingredients might contain high levels of vitamin A. The vitamin A to vitamin D ratio in the fish liver oils is species and fishing area dependent. The ratio range starts with a factor of 0.5 for skipjack liver oil and can even reach an extreme ratio of 119 (pollack liver oil) (Takeuchi et al., 1984). This wide vitamin A to vitamin D ratio range is the reason why fish liver oils often need further processing. In fresh fish products we observe a huge variation in the vitamin D_3_ content per 100 g wet weight (Egaas and Lambertsen, 1979; Takeuchi et al., 1984, 1986; Kobayashi et al., 1995; Mattila et al., 1995a, 1997; Ostermeyer and Schmidt, 2006; Lu et al., 2007; Byrdwell et al., 2013) (Table 1). Large variations in vitamin D_3_ content were found within the same species, but also between the different fish species. Fish obtain their vitamin D_3_ requirements through their diet (Holick, 2003). Therefore, the vitamin D_3_ levels in the zooplankton, the primary food source of fish, or seasonal changes in the zooplankton reservoirs in the different habitats, might be the reasons for the observed fluctuation in the fish product. Interestingly, the weight, the sex, or the age of the fish could not be correlated to the vitamin D_3_ content. Furthermore, no significant correlation between the tissue fat content and vitamin D levels was detected (Mattila et al., 1995a, 1997). Significant differences in vitamin D_3_ content were found between muscle and skin tissues and even more pronounced between muscle and liver tissues (Takeuchi et al., 1986). The 25-hydroxyvitamin D_3_ compound was also detected, though at low concentrations (Takeuchi et al., 1986; Mattila et al., 1995a; Ovesen et al., 2003; Bilodeau et al., 2011).

Wild and sun-dried mushrooms can be a good dietary source of vitamin D_2_ (Mattila et al., 1994, 1999b, 2001; Kobayashi et al., 1995; Teichmann et al., 2007; Phillips et al., 2011). However, the commercially produced mushrooms, e.g., the white button mushroom, do not contain or contain only very low amounts of vitamin D_2_ (Mattila et al., 2001; Teichmann et al., 2007; Phillips et al., 2011). The vitamin D_2_ content in commercially produced mushrooms can be increased by UVB exposure during the culturing or the postharvest process (Mau et al., 1998; Roberts et al., 2008). The concentration of vitamin D in eggs can vary from 0.4 to 12.1 μg (Parrish, 1979; Mattila et al., 1992, 1999a; Bilodeau et al., 2011; Exler et al., 2013), it is in a similar range like offal (Mattila et al., 1995b; Montgomery et al., 2000, 2002). Other animal products like pork, beef, and chicken muscle meat are low in vitamin D content (Mattila et al., 1995b; Montgomery et al., 2000, 2002; Bilodeau et al., 2011; Strobel et al., 2013). By adding vitamin D_3_ into the feed, the vitamin D_3_ content can be increased in muscle and liver of cattle, to 4.6 μg per 100 g of tissue and 99.6 μg per 100 g of tissue, respectively (Montgomery et al., 2004). Milk, unless fortified, has been shown to contain no or very little amounts of vitamin D, whereas in dairy products like butter and cheese the vitamin D content is higher, but in serving size amounts still very low (Kobayashi et al., 1995; Mattila et al., 1995b; Jakobsen and Saxholt, 2009; Trenerry et al., 2011). In general, household cooking seems to have some effect on vitamin D stability depending on the actual foodstuffs and the heating process used (Mattila et al., 1999b; Jakobsen and Knuthsen, 2014).

To meet the vitamin D needs in the countries some states fortify foods. Dairy products are ideal for vitamin D fortification. In Canada vitamin D fortification is mandatory for milk (1 μg/100 ml) and margarine (13.3 μg/100 g) (Health Canada, 2014). In other countries, like the United States, vitamin D fortification is optional for products like milk, breakfast cereals, and fruit juices (Calvo et al., 2004). In the U.S. Department of Agriculture (2013) of the US Department of Agriculture (USDA)'s Nutrient Databank System (Haytowitz et al., 2009), 5036 foods have been determined for their vitamin D content, of which only 259 food items had detectable vitamin D levels. The data showed that per serving only seven fish products had >15 μg vitamin D. All 29 foods that contained between 2.5 μg 15 μg vitamin D per serving were either fortified foods (21) or fish produce (8). Two-thirds of all vitamin D containing foods were far below the 1.0 μg level, whereas 20 percent had even negligible vitamin D content per serving (below 0.1 μg).

Despite the fact that moderate sun exposure of arms and legs in summer for 5–30 min between the hours of 10 a.m. and 3 p.m. twice a week is enough to produce sufficient vitamin D_3_ in the body (Holick, 2007), it is astonishing that many populations that live at these privileged latitudes fail to achieve this goal (Holick and Chen, 2008; Lips, 2010; Wahl et al., 2012; Hilger et al., 2014). During winter time, when vitamin D_3_ production by the sun ceased, adequate vitamin D levels can only be achieved by UVB exposure from indoor tanning units, or by a daily diet of fortified foods or a few selected food items. This restricted list of options to achieve sufficient levels is one of the reasons, why the use of dietary vitamin D supplements has become so popular. It is currently the most applied and secure option to reach adequate vitamin D intake levels (Holick, 2007).

## Vitamin D map, malnutrition, hidden hunger, and nutrition security

An accepted biomarker for the vitamin D status in the general population is to measure the serum concentration of 25-hydroxyvitamin D levels, which is the major circulating form of vitamin D and reflects both dietary vitamin D intake and the endogenous vitamin D production (Lips, 2001, 2007). The serum concentration of 25-hydroxyvitamin D is linked to the serum level of the active hormone 1,25-dihydroxyvitamin D and also to the clinical relevant parathyroid hormone level. Lips has classified the 25-hydroxyvitamin D levels into four stages (Lips, 2001; Lips et al., 2013): severe deficiency (<12.5 nmol/L), deficiency (12.5–25 nmol/L), insufficiency (25–50 nmol/L), repletion (>50 nmol/L). The thresholds for severe deficiency and deficiency are undisputed; however, a controversy has arisen for defining the border between insufficiency and repletion. In 2011, the Institute of Medicine (IOM) suggested a serum level of 50 nmol/L as the value at which 97.5% of the vitamin D needs of the population would be covered (Institute of Medicine, 2011; Ross et al., 2011), whereas, the Endocrine Society (ES) defined it to be higher: 75 nmol/L (Holick et al., 2011). All deficiency levels including insufficiency, as so-called mild deficiency, must be prevented through focused supplementation.

In 2010, the Institute of Medicine (IOM) introduced new dietary reference intake (DRI) values for vitamin D after comprehensive reviewing of more than 1000 high quality research articles to renew thereby their first settings from 1997 (Institute of Medicine, 2011). The DRIs address an adequate nutritional intake of all sources. The IOM has set the dietary allowance (RDA) to 600 IU per day for the general population and at 800 IU per day for persons 70 years and older, whereas 1 IU is the biological equivalent of 0.025 μg vitamin D_3_. The tolerable upper intake level or UL (Upper Level of Intake), which represents the safe upper limit, was set to 4000 IU per day for vitamin D intake (Ross et al., 2011). The new RDAs reflect the scientific outcome from large dietary studies that revealed vitamin D insufficiency (Looker et al., 2002; Zadshir et al., 2005). In 2012, Troesch et al. analyzed the vitamin intake from different dietary surveys that included the German Nutritional Intake Study (Nationale Verzehrstudie II) 2008 (Max Rubner-Institut, 2008), the US National Health and Nutrition Examination Survey (NHANES) from 2003 to 2008 (Centers for Disease Control and Prevention & National Center for Health Statistics, 2009), the UK (The British National Diet and Nutrition Survey, 2003) (Henderson et al., 2003) and the Netherlands (van Rossum et al., 2011), and could confirm that vitamin D is one of the critical vitamins, which intake is below the recommendation (Troesch et al., 2012).

A gap exists between the intake and the recommendation of vitamin D. The chronic insufficient intake of micronutrients like vitamin D without seeing immediate clinical signs is called Hidden Hunger. Hidden Hunger, in particular for vitamin D, is more prevalent in the populations of the developed countries as anticipated (Biesalski, 2013). Hidden Hunger is a threat for the nutrition security for a given country. Nutrition security mandates sufficient micronutrients in an adequate food supply and is required to safeguard an optimal nutritional status of a population.

**Figure 1:**
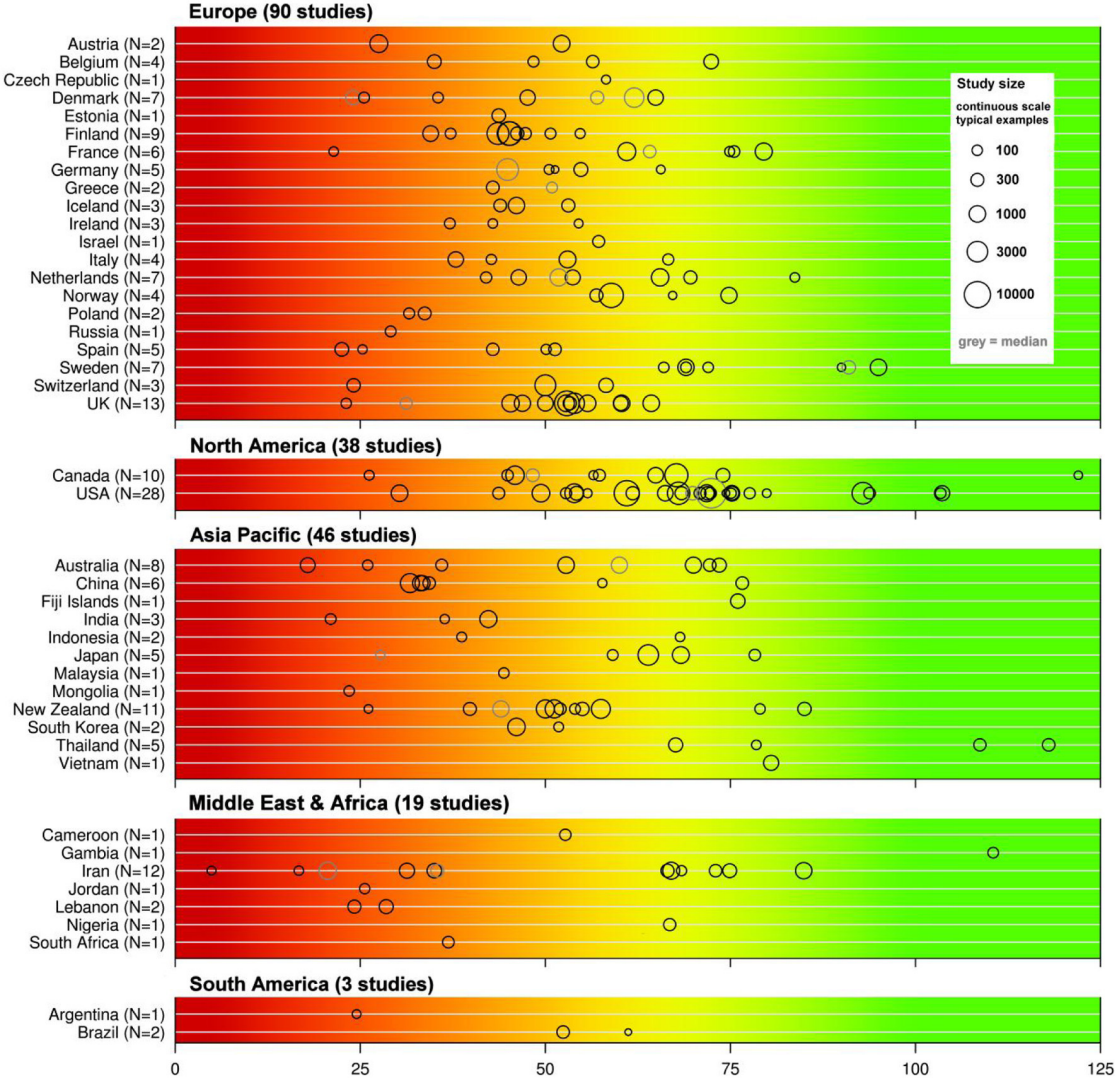
**Overview of published 25-hydroxyvitamin D mean/median values by countries (modified from Hilger et al., 2014)**. The color trend from red, yellow to green shown above the graphical diagram represents the current uncertainty around the definition of 25-hydroxyvitamin D_3_ serum thresholds starting from severe deficiency (red), deficiency, insufficiency to total repletion (green). The reported means are shown as black circles, studies that reported medians are given in gray circle. The study size is indicated by the circle size. Mean/median values falling within the intensely red zone are most consistent with severe vitamin D deficiency.

Many groups have identified vitamin D deficiency or insufficiency to become a public health problem worldwide (Holick, 2007; Holick and Chen, 2008; Mithal et al., 2009; Lips and Van Schoor, 2011; Wahl et al., 2012; Hilger et al., 2014). Mithal et al. (2009) described in their global report that most populations do not achieve a desirable vitamin D status and particular people at risk and elderly people suffer from vitamin D deficiency. In two reports, the International Osteoporosis Foundation (IOF) and its partners published the global vitamin D status map (Wahl et al., 2012; Hilger et al., 2014). The vitamin D map was based on a systematic review of the worldwide vitamin D levels, using all available publications published between 1990 and February 2011 (Hilger et al., 2014). Eligible studies include 168,389 participants from the general populations throughout the world where the mean or median serum 25-hydroxyvitamin D levels were measured. Studies included had a cross-sectional design or were based on a population based cohorts. The analysis identified nearly 200 studies from 44 countries, whereas only half of the studies were included in the global vitamin D status map as 50.2% of the studies were not representative for the target populations. Figure 1 shows the global vitamin D status map listed by countries and by continents. The largest numbers of studies were performed in Europe, followed by North America and Asia-Pacific. Available data from Latin America and even more from Africa are limited. Results of this review showed that 6.7% of the population were vitamin D deficient (mean 25-hydroxyvitamin D values <25 nmol/L), 37.3% were vitamin D insufficient according to IOM (mean values below 50 nmol/L) and 88.1% of the population showed an insufficient vitamin D status according to the ES (mean values below 75 nmol/L). No significant differences were found for gender or age, when looking at the worldwide data, but some regional differences could be identified (Hilger et al., 2014). The 25-hydroxyvitamin D serum levels were higher in Europe and the US, when compared to Middle East and Africa. This might be due to the vitamin D food fortification programs in North America (Prentice, 2008). Furthermore, the systematic analysis revealed that institutionalized elderly were more at risk to have low 25-hydroxyvitamin D levels in Europe and Asia/Pacific. The compared non-institutionalized elderly group showed higher levels, possibly due to spending more of time outdoors. The group of institutionalized elderly is therefore at high risk to become vitamin D deficient. Further research is needed to inform public health policy makers to reduce the risk for potential health consequences of low vitamin D status.

In the past few years the national recommendations for dietary vitamin D were adjusted in several countries; they are not harmonized across the European Union yet and vary from 200 to 800 IU. The higher recommendations for dietary vitamin D intake are increasingly being suggested in government documents, position statements and clinical practice guidelines for bone health. In 2008, the US Food and Drug Administration updated the health claim for the prevention of osteoporosis by including vitamin D to the consumption of calcium (Food and Drug Administration, 2008). In 2008, the American Academy of Pediatrics also reacted and issued an update of their guidelines for vitamin D intake and rickets prevention (Perrine et al., 2010). They doubled the recommended dose of vitamin D for children to 400 IU per day, beginning in the first few days of life and continuing throughout adolescence. In 2010, the Institute of Medicine (IOM) released the revised Dietary Reference Intakes (DRI's) for calcium and vitamin D and tripled the recommendations for vitamin D intakes to 600 IU per day for children and all adults up to age 69 years (Institute of Medicine, 2011). The IOM stated that there was insufficient evidence to make recommendations for non-skeletal benefits.

In 2012, the German, Austrian, and Swiss Nutrition Societies raised the recommended vitamin D intake to 800 IU per day, in case of absent UVB exposure, for all age groups starting from 1 year of age (German Nutrition Society, 2012). Furthermore, key opinion leaders are increasingly recommending higher daily intakes for vitamin D, between 800 and 1000 IU or even higher for people at risk or older adults. The recent statement by the IOF and the guidelines by the US ES suggest that higher vitamin D doses would be needed to achieve the desirable 25-hydroxyvitamin D serum level of 75 nmol/L for people at risk or older individuals.

Increasing the vitamin D levels in the population would also ameliorate health economics. Grant and colleagues calculated the benefit of increasing vitamin D levels to reduce the economic burden of diseases (Grant et al., 2009). A rise in the vitamin D serum level of all Europeans to 40 nmol/L would reduce the economic burden of different diseases and could save health care costs of up to 16.7%. Besides reducing the economic costs, vitamin D intake could in addition also reduce mortality rates and maintain a longer healthy life style.

## Nutritional research to address and understand vitamin D insufficiency

Vitamin D deficiency is undoubtedly linked to severe consequences in the growing child by causing incomplete mineralization of the bone and in the adult accounting to wasteful osteomalacia. In the vitamin D insufficiency stage, this severity gets gradually less, but the outcome remains unchanged. Besides the established and accepted functional skeletal health relationship, more and more evidence is accumulating for falls (Pfeifer et al., 2000, 2009; Bischoff et al., 2003; Flicker et al., 2005; Broe et al., 2007; Prince et al., 2008; Bischoff-Ferrari et al., 2009) and physical performance (Bischoff-Ferrari et al., 2004; Houston et al., 2011; Ceglia et al., 2013; Redzic et al., 2013; Sohl et al., 2013; Tieland et al., 2013), which has been recognized by a health claim of the European Food and Safety Authority in 2011: “Vitamin D may reduce the risk of falling. Falling is a risk factor for bone fractures.” This health claim is targeting men and women 60 years of age and older and the dose required is a daily consumption of 800 IU vitamin D, which can come from all sources. Further emerging vitamin D health relationships include physiological parameters like improved immune response (Baeke et al., 2010; Schwalfenberg, 2011; Hewison, 2012; White, 2012), improved respiratory health(Berry et al., 2011; Charan et al., 2012; Choi et al., 2013; Hirani, 2013) possibly also relate to reduced tuberculosis incidence (Nnoaham and Clarke, 2008; Martineau et al., 2011; Mitchell et al., 2011; Coussens et al., 2012; Salahuddin et al., 2013; Huaman et al., 2014); and reduced risk to develop autoimmune diseases like multiple sclerosis (Solomon and Whitham, 2010; Cantorna, 2012; Dobson et al., 2013) or type 1 diabetes (Hypponen et al., 2001; Holick, 2003; Ramos-Lopez et al., 2006; Baeke et al., 2010; De Boer et al., 2012; Dong et al., 2013; Van Belle et al., 2013). In chronic, non-communicable diseases, vitamin D deficiency is being discussed to possibly ameliorate the incidence of some neoplastic diseases like colorectal, lung, prostate, and breast cancers (Ng et al., 2008; Rosen et al., 2012; Welsh, 2012; Cheng et al., 2013); cardiovascular diseases (CVDs) including hypertension, myocardial infarction, stroke (Forman et al., 2007; Giovannucci et al., 2008; Gardner et al., 2011; Bischoff-Ferrari et al., 2012; Tamez and Thadhani, 2012; Karakas et al., 2013; Pilz et al., 2013a; Schroten et al., 2013); life-style diseases like obesity and type 2 diabetes (Pittas et al., 2007; González-Molero et al., 2012; Khan et al., 2013; Pilz et al., 2013b; Schottker et al., 2013; Tsur et al., 2013; Van Belle et al., 2013; Bouillon et al., 2014); diseases related to the decline in sight function including age-related macular degeneration (Parekh et al., 2007; Millen et al., 2011; Lee et al., 2012); and neurological disorders including Alzheimer and Parkinson disease (Buell and Dawson-Hughes, 2008; Annweiler et al., 2012; Eyles et al., 2013; Zhao et al., 2013). One may wonder about the width of possible implications being looked at, but considering the more than 1000 genes which vitamin D is regulating through the VDR (Carlberg and Campbell, 2013), this may actually not be a surprise. To determine the potential role of vitamin D supplementation in the prevention or treatment of chronic non-skeletal diseases notwithstanding, large-scale clinical trials are demanded. In this respect for the nutrition field, four new large-scale ongoing long-term supplementation studies are expected to deliver results in near future (Table 2). The two very large studies, VITAL trial (*n* = 20,000) and FIND study (*n* = 18,000), are meant to deliver clinical evidence for the effect of vitamin D_3_ on cancer, CVD and diabetes outcomes. The two smaller trials, CAPS and DO-HEALTH, each having more than 2,000 participants are including cancer, infections, fractures, hypertension, cognitive function, and physical performance outcomes. In all four studies the placebo group will produce vitamin D_3_ in the skin and will possibly consume vitamin D through food, and therefore this will narrow the vitamin D serum level gap between the placebo and treatment groups. It remains to be seen whether the applied supplementation doses (2000 IU and 1600 IU, 3200 IU) will be sufficient to see a clear difference between the treatment and the control groups. An open likelihood will remain for the placebo group potentially obtaining sufficient vitamin D_3_ (600–800 IU) levels that are considered to be sufficient for skeletal effects. In such a case only an incremental increase of an additional ~1000 IU can be considered as the effective dose, for which no power calculation was available at the time before study begun. In light of such a situation, it will be of interest whether the micronutrient triage theory of Bruce Ames can be validated with vitamin D_3_ (Ames, 2006; McCann and Ames, 2009). The triage theory postulates, as a result of recurrent shortages of micronutrients during evolution, that the body has selected and developed a metabolic rebalancing response to shortage. These rebalancing favored micronutrient-needs for short term survival, while those only required for long-term health were starved. In the case of the micronutrient vitamin D_3_, calcium and bone metabolism can be considered to be secured with highest priority, therefore, it might be speculated that the 600–800 IU intake would satisfy this vitamin D_3_ serum level threshold. For the chronic non-skeletal diseases however, which have only secondary priority in an evolutionary perspective, higher serum vitamin D_3_ levels would be required. The ongoing four vitamin D_3_ studies that have chronic diseases as their main outcomes and use nutritionally relevant ~2000 IU are therefore well-suited to address whether the triage theory holds also true for the micronutrient vitamin D_3_.

**Table 2:** **List of ongoing large nutritional vitamin D_3_ supplementation trials (>2,000 subjects) using nutrition-related daily vitamin D_3_ doses (1,600–3,200 IU)**.

**Acronym**	**Name, clinical trial identifier**	**Principal investigator**	**Place**	**Participants**	**Dose**	**Duration**	**Main outcomes**	**Results expected**	**Web link**
CAPS	Clinical Trial of Vitamin D_3_ to Reduce Cancer Risk in Postmenopausal Women NCT01052051	Joan Lappe, Creighton University	USA	2,332, healthy postmenopausal women: 55+	2,000 IU D_3_ (and 1,500 mg calcium) daily	5 years	All cancers	2015	http://clinicaltrials.gov/ct2/show/NCT01052051?term=NCT01052051&rank=1
VITAL	Vitamin D and Omega-3 Trial NCT01169259	JoAnn E. Manson, Brigham and Women's Hospital	USA	20,000, men: 50+ women: 55+	2,000 IU D_3_, daily omega-3 fatty acids	5 years	Cancer, Cardiovascular disease	2017	http://clinicaltrials.gov/show/NCT01169259
DO-HEALTH	Vitamin D3—Omega3—Home Exercise—Healthy Ageing and Longevity Trial NCT01745263	Heike Bischoff-Ferrari, University Zürich	8 European Cities	2,152, 70+	2,000 IU D_3_ daily omega-3 fatty acids	3 years	Infections, Fractures, Blood pressure, Cognitive function, Lower extremity function	2017	http://clinicaltrials.gov/ct2/show/NCT01745263?term=bischoff-ferrari&rank=1;
FIND	Finnish Vitamin D Trial NCT01463813	Tomi-Pekka Tuomainen, University of Eastern Finland	Finland	18,000 men: 60+, women: 65+	1,600 IU D_3_ daily or 3,200 IU D_3_ daily	5 years	Cancer, Cardiovascular disease Diabetes	2020	http://clinicaltrials.gov/show/NCT01463813

Vitamin D_3_ once in the blood immediately binds to the vitamin D-binding protein (DBP) and gets transported into the liver (Holick, 2007). The first hydroxylation at position 25 generates the major circulating metabolites 25-hydroxyvitamin D_3_. This metabolite circulates throughout all organs and undergoes hydroxylation at position 1, which occurs mainly in the kidney, but also in other organs, to form 1,25-dihydroxyvitamin D_3_, the active hormone. Besides the major circulating metabolite 25-hydroxyvitamin D_3_ and the hormonally active metabolite 1,25-dihydroxyvitamin D_3_, more than 35 additional vitamin D_3_ metabolites are formed by the body (Bouillon et al., 1995; Norman et al., 2001). It is speculated that they might be intermediates in the catabolism of 1,25-dihydroxyvitamin D_3_. The human body has evolved many CYP enzymes and invests energy to form these additional 35 vitamin D_3_ metabolites, whether this is for the purpose to catabolize 1,25-dihydroxyvitamin D_3_, remains still to be answered. More appealing is the theory that these metabolites are formed to fulfill yet unknown functions of vitamin D_3_. This perspective could potentially also account to the pleiotropic non-skeletal health benefits reported by the many vitamin D intake studies. For some of the vitamin D_3_ metabolites like the 24*R*,25-dihydroxyvitamin D_3_ potential function was explored *in vitro* (Norman et al., 2002).

The 24*R*,25-dihydroxyvitamin D_3_ has been shown to be an essential hormone in the process of bone fracture healing. The 24*R*,25-dihydroxyvitamin D_3_ most likely initiates its biological responses via binding to the ligand binding domain of a postulated cell membrane receptor VDR_mem24,25_, similar to the better studied, but still not cloned cell membrane receptor for 1,25-dihydroxyvitamin D_3_, VDR_mem1,25_ (Norman et al., 2002). From the nutritional point of view, it will be of interest to investigate the function of the all vitamin D_3_ metabolites and relate the function to the level of vitamin D_3_ intake to secure the health benefit according to the triage theory.

According to the current knowledge, the vitamin D endocrine system is funneled through the biologically most active metabolite 1,25-dihydroxyvitamin D_3_ that is mainly produced in the kidney, but also in other organs (Bouillon et al., 2013). Mechanistically 1,25-dihydroxyvitamin D_3_ binds the VDR directly on a DNA sequence, the 1,25-dihydroxyvitamin D_3_ response element (VDRE), in the regulatory region of primary 1,25-dihydroxyvitamin D_3_ target genes (Carlberg and Campbell, 2013). The VDR forms together with the retinoid X receptor or putative other transcription factors a heterodimer on the VDRE, recruiting tissue-specific transcriptional co-activators and regulates through a conformational change upon 1,25-dihydroxyvitamin D_3_ binding the downstream gene. The VDR is widespread in more than 30 tissues (Bouillon et al., 1995) and may trigger expression of more than 1000 genes through 1,25-dihydroxyvitamin (Carlberg et al., 2013; Hossein-Nezhad et al., 2013). The regulation of tissue-specific gene expression by 1,25-dihydroxyvitamin D_3_ is of high interest, as it guides us toward the better understanding of the mechanistic action of vitamin D_3_ in the different tissues. The gained knowledge from the mechanistic studies can help to design smaller and more focused nutritional intervention RCTs to answer whether vitamin D contributes to a specific health benefit of interest. In this respect the GeneChip-based transcriptomics methodology using high-density microarrays demonstrated the expression of genes in a variety of important functions of more than 100 different pathways that could be linked to vitamin D deficiency (Bossé et al., 2007; Tarroni et al., 2012; Hossein-Nezhad et al., 2013). The development of chromatin immunoprecipitation (ChiP) methodology linked to site-specific PCR amplification of the VDR bound genomic DNA fragment, and later the methods using tiled microarrays (ChiP-chip) applying the first unbiased genome-wide approach, which then was followed by the massive parallel NGS sequencing approach of the immunoprecipitated DNA segments, opened up new avenues to investigate 1,25-dihydroxyvitamin D_3_ target genes in selected tissues (Ramagopalan et al., 2010; Heikkinen et al., 2011; Carlberg et al., 2012, 2013; Pike et al., 2014). In an elegant study, Carlberg et al. identified in samples of 71 pre-diabetic individuals of the VitDmet study changes in serum 25-hydroxyvitamin D_3_ concentrations that were associated to primary vitamin D target genes (Carlberg et al., 2013). Based on their finding the authors proposed the genes CD14 and THBD as transcriptomics biomarkers, from which the effects of a successful vitamin D_3_ supplementation can be evaluated. These biomarkers are potentially suitable for displaying the transcriptomics response of human tissues to vitamin D_3_ supplementation.

Epigenetic alterations of the genome refer to heritable and modifiable changes in gene expression that are not affecting the DNA sequence. They may be inherited as Mendelian, non-Mendelian, or environmentally caused traits. One of the 1,25-dihydroxyvitamin D_3_ induced epigenetic modification was shown for the hypo-methylating effect on the osteocalcin promoter (Haslberger et al., 2006). 1,25-Dihydroxyvitamin D_3_ was associated with the demethylation of the osteocalcin promoter and induced the osteocalcin gene expression. The activity of VDR can be modulated by epigenetic histone acetylation. The VDR alone or in concert with other transcription factors can recruit histone-modifying enzymes like histone acetyl transferases (HATs) or histone deacetylases (HDACs) and epigenically direct transcriptional expression of downstream genes (Burrell et al., 2011; Karlic and Varga, 2011; Sundar and Rahman, 2011; Hossein-Nezhad et al., 2013). The trans-generational epigenetic inheritance of vitamin D_3_ triggered epigenome modification is not fully explored, however maternal vitamin D deficiency has been discussed with adverse pregnancy outcomes or potential susceptibility for diseases (Burrell et al., 2011; Hossein-Nezhad and Holick, 2012). For future nutritional research it would be of great value to identify and validate epigenetic biomarkers that could serve as risk assessment tool for vitamin D insufficiency related susceptibility to develop a disease later in life.

Variations in vitamin D status have been shown to be related to inheritance. The disparity of vitamin D levels according to ethnicity given skin pigmentation is well-established (Cashman, 2014; Ng et al., 2014). Dark skinned population individuals have compared to Caucasian descendants almost one-half the serum concentrations of 25-hydroxyvitamin D (Nesby-O'dell et al., 2002). From twin studies it has been estimated that the heritability of genetic regulation of vitamin D levels to be between 23 and 80% (Dastani et al., 2013). In addition, large-scale genetic association studies using linkage disequilibrium analysis have identified genetic loci correlating with serum vitamin D level within five candidate genes (Dastani et al., 2013). The identified SNPs are within the 1alpha-hydroxylase of 25-hydroxyvitamin D (CYP27B1) gene, the 25-hydroxylase of vitamin D (CYP2R1) gene, the vitamin D carrier protein (GC) gene, the VDR gene, and the cytochrome P-450 (CYP24A1) gene coding for an enzyme that inactivates 1,25-dihydroxyvitamin D. It is important to note that replication studies in separate populations have to follow to verify the validity of the identified SNPs. The SNP information will provide the additional guidance toward a personalized nutritional advice to reach a sufficient vitamin D status.

## Conclusion and future perspectives

In the recent years the knowledge about vitamin D and its implications have extended far beyond its classical role in bone health in either fields of basic research as well as in human trials. In particular, the evidence for the role of vitamin D in reducing the risk of fractures as well as decreasing the risk for falling is convincing and authorities have responded to it. Besides a health claim issued by the EFSA on the risk reduction for falling the dietary intake recommendations have been significantly increased in several countries such as the US and in Europe (Austria, Germany, Switzerland). A number of other countries around the globe are in the process of establishing new dietary intake recommendations as well. It turns out that on average a daily intake of 600–800 IU vitamin D appears to be required to meet fundamental needs of the human body, for specific applications higher daily intakes may be necessary, which will become clearer as the results of a number of ongoing clinical studies will become available.

The obvious question to answer is: do people obtain the recommended amounts of vitamin D? The diet is typically only a minor vitamin D source as only few food items contain relevant amounts of vitamin D, such as fatty sea fish. The primary vitamin D source for humans is the vitamin D synthesis in the skin from vitamin D precursors by the sunlight—provided the skin is sufficiently exposed to strong enough sun radiation. Several groups have reviewed the published results on 25-hydroxyvitamin D serum levels the established marker of the vitamin D status, showing that low 25-hydroxyvitamin D levels are found in many cohorts around the world. A recent systematic review of the global vitamin D status (Hilger et al., 2014) showed that 6.7% of the overall populations reported deficient 25-hydroxyvitamin D levels below 25 nmol/L, 37% had 25-hydroxyvitamin D levels below 50 nmol/L, and only 11% were above 75 nmol/L, which is considered an adequate status by the IOF and the ES. So a very important task ahead of us is to find efficient ways to improve the vitamin D status on the population level, be it by dietary means, food fortification, or dietary supplements.

In addition, it will be very important to gather sound and convincing evidence for the many additional implicated health benefits of vitamin D besides the ones that already reached a health claim status and to see which of them will actually hold up. This will require appropriate human studies on the one hand, and also involve the appropriate use of the novel experimental approaches like nutrigenomics, nutrigenetics, and nutriepigenetics on the other hand. In conclusion, the evidence we have for vitamin D in human health is exciting, however we have to make sure that appropriate measures are taken to improve the vitamin D status to the levels required to be beneficial for human health. In future, we will also need to further apply, exploit and invest in novel, innovative and break-through technologies in the vitamin D research to understand the underlying mechanisms by which vitamin D is exerting so many effects in the human body, which is knowledge needed to the purpose to obtain and secure optimal public health through nutrition.

### Conflict of interest statement

The authors are employees of DSM Nutritional Products Ltd. and declare to have no conflict of interest.
